# Development of a composite score to predict Lewy body pathology burden

**DOI:** 10.1002/alz.092056

**Published:** 2025-01-03

**Authors:** Parichita Choudhury, Kewei Chen, Nan Zhang, Cécilia Tremblay, Andrew Ho, Christine Belden, Charles Adler, Holly Shill, Shyamal Mehta, Erika Driver‐Dunckley, David Shprecher, Geidy E Serrano, Thomas G. Beach, Eric M. Reiman, Alireza Atri

**Affiliations:** ^1^ Banner Sun Health Research Institute, Sun City, AZ USA; ^2^ Arizona State University, Tempe, AZ USA; ^3^ University of Arizona, Tucson, AZ USA; ^4^ Banner Alzheimer’s Institute, Phoenix, AZ USA; ^5^ Mayo Clinic, Scottsdale, AZ USA; ^6^ Banner Sun Health Research Institute, Phoenix, AZ USA; ^7^ Barrow Neurological Institute, Phoenix, AZ USA; ^8^ Mayo Clinic Arizona, Scottsdale, AZ USA; ^9^ Center for Brain/Mind Medicine, Department of Neurology, Brigham and Women’s Hospital and Harvard Medical School, Boston, MA USA

## Abstract

**Background:**

Lewy body (LB) diseases can present with overlapping prodromal, cognitive, motor, autonomic or neuropsychiatric symptoms. Intuitively, greater symptom severity should correlate with greater pathological burden, but this has not been consistently shown. LB pathology does not translate to clinical expression in Incidental LB disease. While several composite scores/toolkits utilize overlapping schemes for diagnosis, a composite data‐derived score to predict LB pathologic burden is lacking. We aimed to utilize an autopsy‐confirmed cohort, and validated, brief, and standardized clinical, functional, pathologic assessments, and machine learning (ML)/quantitative modeling techniques to identify clusters and importance rankings among assessments/measures to predict LB pathologic severity and density.

**Method:**

A total of 234 subjects with neuropathological finding of LB at autopsy in the Arizona Study of Aging and Neurodegenerative Disorders (AZSAND) who were classified as Cognitively Unimpaired or Mild Cognitive Impairment after their first study clinical evaluation were included. Of these, 145 subjects had complete scores for 32 of 46 measures (clinical, cognitive, behavioral and functional) that were utilized as predictors of LB pathology. Olfactory function was assessed using the University of Pennsylvania Smell Identification Test (UPSIT). LB severity was assessed by the Unified Staging System for Lewy Body Disorders (USSLB). Several ML algorithms and quantitative prediction models were explored and compared: artificial neural network (ANN), partial least square regression (PLSR), support vector regression (SVR), relevance vector regression (RVR) and ensemble forest regression (EFR) with leave‐one‐out (LOO) scheme. Game‐theory based Shapley methods assessed the impact, including rankings, consistency and magnitude, of model predictors.

**Result:**

RVR predicted aggregate LB density with large effect size (R^2^ = 0.691, p<0.00001). ANN predicted USSLB severity stage with large effect size (R^2^ = 0.74, p<2.5e‐43). All other ML algorithms/models provided substantial prediction. Across all models, UPSIT was the most influential predictor (>65%), followed by Controlled Oral Word Association Test (COWAT) and age.

**Conclusion:**

These preliminary and exploratory results support the utilization of ML techniques/models to assess LB pathologic burden with key measures collected in relatively small samples. UPSIT was consistently ranked highest impactful among clinical and functional measures/predictors. Integrated together in ML/data‐derived composites, UPSIT, COWAT and other clinical characteristics may be of antemortem utility to predict USSLB stages.

